# Case Report: Giant insulinoma, a very rare tumor causing hypoglycemia

**DOI:** 10.3389/fendo.2023.1125772

**Published:** 2023-05-10

**Authors:** Georges Tarris, Alexia Rouland, Kévin Guillen, Romaric Loffroy, Anne-Cécile Lariotte, Patrick Rat, Benjamin Bouillet, Haingo Andrianiaina, Jean-Michel Petit, Laurent Martin

**Affiliations:** ^1^ Department of Pathology, University Hospital of Dijon, Dijon, France; ^2^ Department of Endocrinology and Diabetology, University Hospital of Dijon, Dijon, France; ^3^ Department of Radiology, University Hospital of Dijon, Dijon, France; ^4^ Department of Digestive and Bariatric Surgery, University Hospital of Dijon, Dijon, France

**Keywords:** giant insulinoma, pancreas, neuroendocrine tumors, hypoglycemia, pathology

## Abstract

Insulinomas, with an incidence of 4 cases per million individuals per year, remain amongst the most frequent functional neuroendocrine tumors. The usual diameter of insulinomas usually remains under 3 cm of major axis. However, 44 exceptional cases of “giant insulinomas”, have been reported worldwide, generally exceeding 9 cm in major axis. In this article, we report the case of a 38-year-old woman whom suffered from chronic hypoglycemia despite treatment with diazoxide. Abdominal CT-scan revealed a 88 x 73 mm mass located at the tail of the pancreas. Following surgical excision, histopathological analysis confirmed G1 neuroendocrine tumor, with focal cytoplasmic expression of insulin in tumor cells. After a 16-month follow-up period, the patient didn’t address any specific complaint, and no disease recurrence and/or metastasis were observed. A ^68^Ga-DOTATATE-PET scan was performed 6 months after surgery, which came back normal. Genetic evaluation has not been performed in our patient. The physiopathology of giant insulinomas remain unexplained, however with possible relationship with type 1 multiple endocrine neoplasia, sporadic somatic *YY1* mutations and possible transformation of bulky non-functional pancreatic neuroendocrine tumors to a functional phenotype, with slow insulin secretion. While giant insulinomas remain rare in the literature, multicentric genetic analysis of tumor samples might reveal unique features of this rare subtype of neuroendocrine pancreatic tumors. Insulinomas of large size tend to have greater malignancy and higher rates of invasiveness. Careful follow-up, especially for liver and lymph node metastases, must be performed using functional imaging techniques to avoid disease relapse.

## 1 Introduction

Insulinomas are neuroendocrine tumors with very low incidence rates ranging between 1 to 4 cases per million inhabitants per year, with a predilection for females in the fourth or fifth decade (1). Autopsy studies suggest much higher rates of insulinoma, demonstrating the current underdiagnoses of these tumors (2). Insulinomas can be sporadic or associated with genetic predispositions such as type 1 multiple endocrine neoplasia (MEN1), with 11q13 loss of heterozygosity (3). The MEN1 syndrome is characterized by the association of primary hyperparathyroidism, pituitary adenomas and gastric/pancreatic tumors such as gastrinomas or insulinomas (4). In very rare cases, insulinomas can be associated with tuberous sclerosis or neurofibromatosis type 1 (NF1) (5, 6).

As a general rule, most patients with insulinoma present with hypoglycemia, however diagnosis can be challenging in smaller tumors, given that the average size of these tumors is no greater than 3 cm of diameter in 95% of cases (1, 7). In some cases, the “Whipple’s triad” including hypoglycemia, neuroglycopenic symptoms and relief of symptoms following glucose intake might constitute a “red flag” for the diagnosis of insulinoma (8). The diagnostic confirmation of insulinoma is initially performed upon the analysis of blood markers, including elevated insulin, pro-insulin and C-peptide levels concomitantly to decreased blood glucose levels (9, 10).

Management of insulinomas include curative surgery, and medical treatment such as the use of diazoxide or somatostatin analogs (e.g., octreotide) to alleviate symptoms and induce tumor regression (9, 10). Other medical options include the use of mammalian target of rapamycin (mTOR) inhibitors (e.g., everolimuis, especially in malignant insulinomas) or tyrosine kinase inhibitors (such as sunitinib), as the growth of insulinomas involve the mTOR and tyrosine kinase receptor pathways, through the involvement of IGF1 (11, 12). More recently, distant metastases have been successfully treated through the use of peptide receptor radionuclide therapy (PRRT) (13).

Insulinomas are commonly considered as benign tumors, however malignancy could be associated with tumors of greater size with higher metastatic rates (14, 15). Despite the higher probability of metastasis and poorer outcome, malignant insulinomas show variable clinical outcomes and survival rates, mainly depending on tumor biology rather than surgical and/or medical treatment (16). Malignant insulinomas tend to be of greater size than benign insulinomas, including “giant” insulinomas which also tend to behave more aggressively (14). A cohort study by Sada et al. showed that the average size of benign insulinomas was measured around 1 cm, in comparison to malignant insulinomas with an average size of 4 cm (14).

In this article, we report the case of a 38-year-old female patient suffering from a non-metastasizing insulin-secreting giant pancreatic neuroendocrine tumor (PanNET) and discuss the pathogenesis and current knowledge regarding giant insulinomas.

## 2 Case description

A 38-year-old female patient was admitted to the University Hospital of Dijon for multiple episodes of malaise, fatigue and fainting at home for the last 3 months. The episodes of fainting were experienced by the patient before noon or by the end of the afternoon, usually before meals. Increased food intake at specific timepoints before meals alleviated the symptoms. Past medical history includes appendectomy and use of a contraceptive intrauterine device. Family history includes prostate cancer in one of the grandfathers. The patient was not on any specific medication by time of the diagnosis, however regular tobacco use was noted.

**Figure 1:**
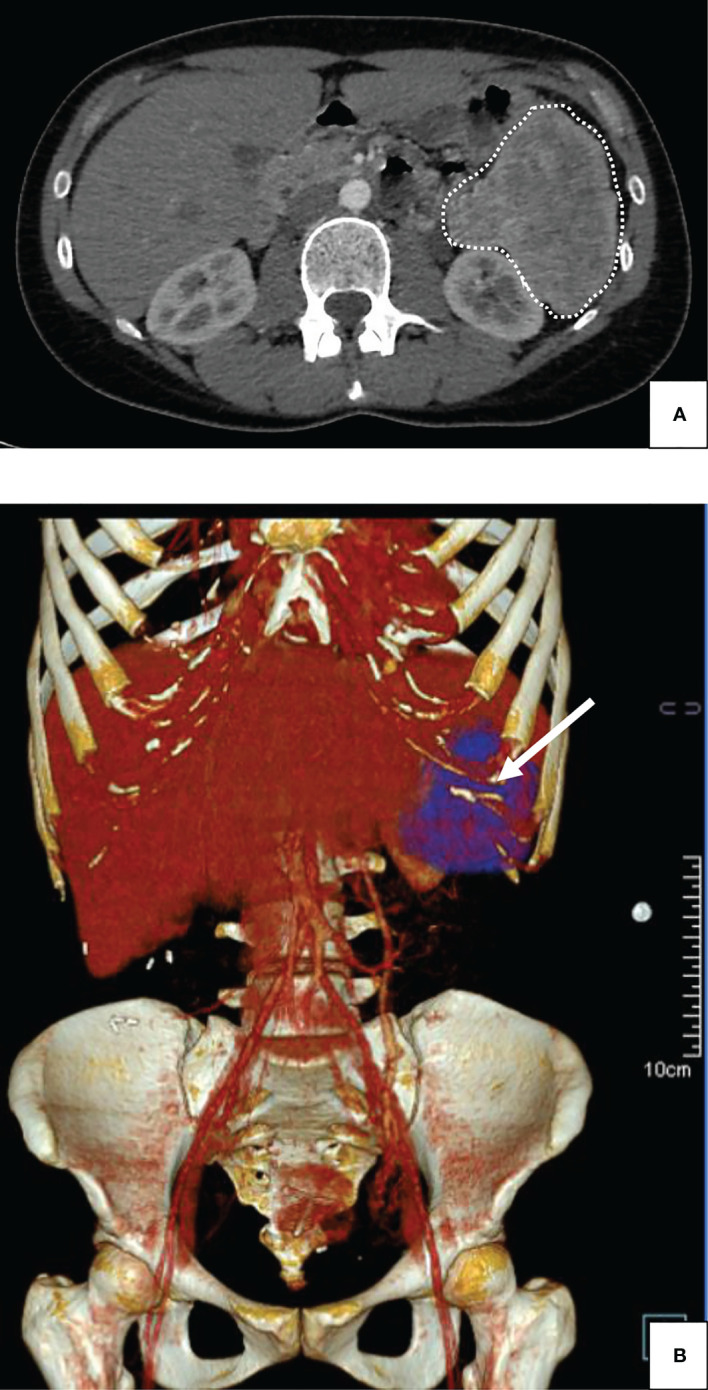
Abdominal injected CT-scan of a giant insulinoma in a 38-year-old female patient suffering from severe hypoglycemia. **(A)** Plain Computed Tomography scan (CT-scan); horizontal cut: the 88 mm-wide mass was observed in the left hypochondrium, without invasion of adjacent structures (dashed lines). **(B)** Plain Computed Tomography scan (CT-scan); tridimensional reconstruction: The absence of organ invasion was confirmed using three-dimensional reconstruction for computed tomography (arrow).

**Figure 2:**
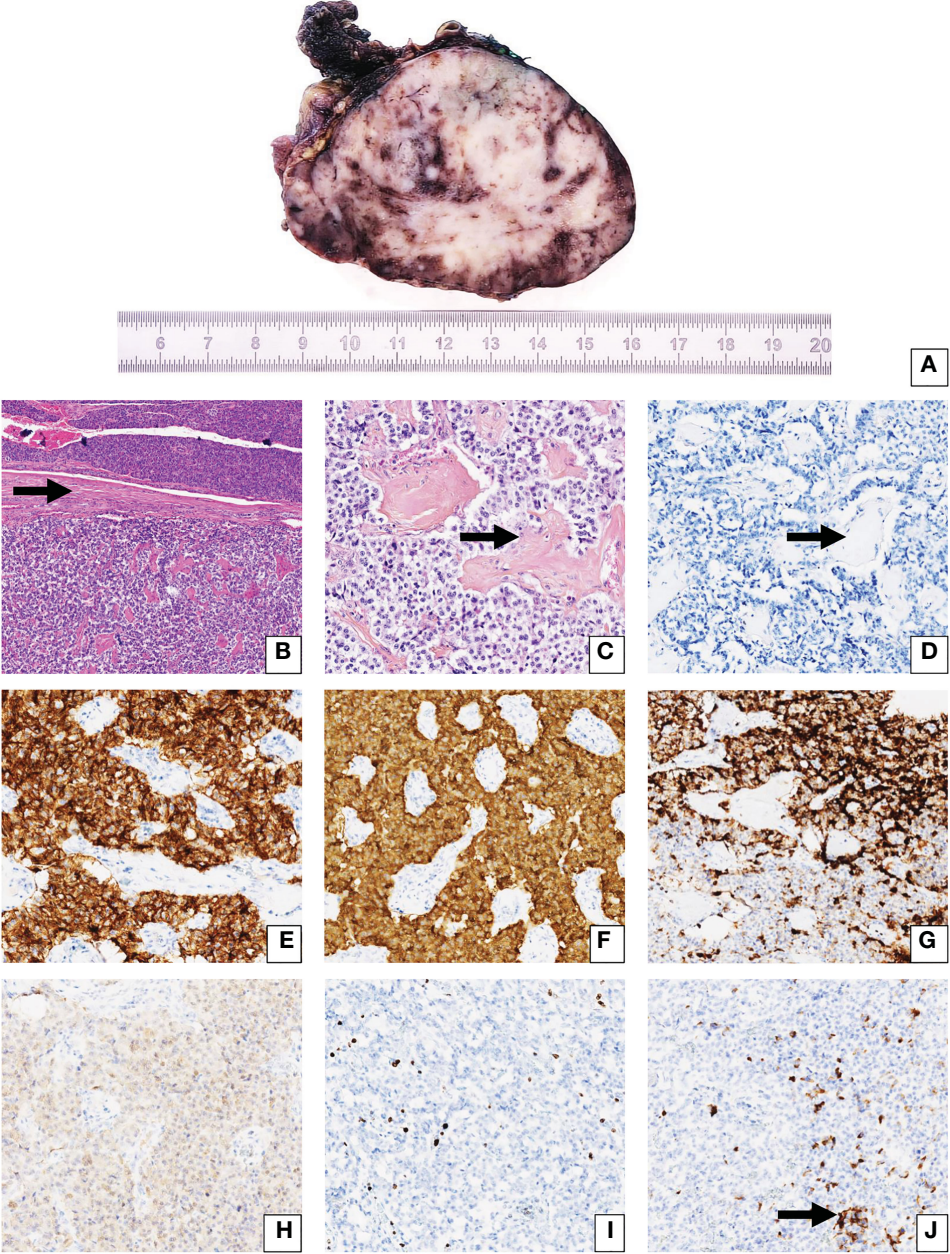
Gross description and histopathological analysis of a giant insulinoma in a 38-year-old female patient suffering from severe hypoglycemia. Positive detection of antibodies is indicated by brown staining. **(A)** (Photography – horizontal cut-section): Gross examination of the tumor following surgical resection and formalin fixation during 48 hours. The encapsulated tumor of approximately 80 mm of diameter shows well-defined borders and a homogenous whitish-grey appearance at cut-section with several hemorrhagic foci. **(B)** (HES, x 200): Histopathological analysis shows a monophasic proliferation of nests and cords of basophilic cells, organized around numerous vascular slits and inside a moderately thick fibrous capsule (arrow). No atypia, necrosis or capsular invasion were noted. The mitotic index was estimated at approximately 1 mitosis**/**10 hpf. **(C)** (HES, x 400): Endocrine cells were also surrounded by a hyaline stroma (arrow). **(D)** (Congo Red, x400) The hyaline stroma was negative for Congo Red staining (arrow). **(E)** (CD56, x 400): Tumor cells showed intense diffuse cytoplasmic staining. **(F)** (Synaptophysin, x 400): Tumor cells showed intense diffuse cytoplasmic staining. **(G)** (Chromogranin A, x 400): Tumor cells showed intense heterogeneous cytoplasmic staining. **(H)** (Neuron-Specific Enolase, x 400): Tumor cells showed weak diffuse cytoplasmic staining. **(I)** (Ki-67, x 400): The Ki-67 proliferation index was estimated around 1**%**. **(J)** (Insulin, x 400): Few scattered cells were positive for insulin immunostaining (arrow).

The patient was soon referred to the Department of Endocrinology, as fasting capillary blood glucose measurements (CBGM) were as low as 1.99 mmol/L (35.8 mg/dL). At admission, the patient weighted 58.2 kg. Concomitant fasting insulin and C-peptide assays revealed inappropriate insulin secretion, with C-peptide at 2.01 ng/mL, pro-insulin at 91.56 pmol/L and insulin levels at 13.6 mIU/L. Liver function tests, hemoglobin (13 g/dL), albumin levels (37 g/L), creatinine (63 µmol/L) and calcium (2.44 mmol/L) levels were otherwise normal. Total bilirubin (26 µmol/L) either conjugated or free was slight increased (normal range: 1.71 to 20.5 µmol/L). CRP levels were low. The TSH (0.65 mIU/L), T4 (16.5 pmol/L), ACTH (29 ng/L) and cortisol levels remained within normal range. The PTH, IGF1 and prolactin levels were within normal range. Genetic analysis for MEN1 gene has not been performed. An abdominal CT-scan was performed, which showed a heterogenous 88 x 73 mm bulky mass of the left hypochondrium located between the tail of the pancreas and the splenic hilum, without evidence of tissue infiltration or peripheral lymph node invasion (**Figure 1**). A Positron-emission tomography (PET) scan was then performed, showing isolated hypermetabolism of the pancreatic mass. Under the high suspicion of an insulin-secreting tumor, the patient was initially treated with 100 mg of Diazoxide three times a day before each meal, which substantially increased fasting CBGM. The patient then underwent laparoscopic distal pancreatectomy with splenectomy for definite curative treatment. The surgical resection specimen was sent to the Department of Pathology (University Hospital of Dijon – France). The surgical resection specimen included the pancreas (35 x 30 x 10 mm) and the spleen (130 x 90 x 45 mm) with the greater omentum (140 x 70 x 5 mm). At gross examination, the tumor was located at the pancreatic tail and measured approximately 90 x 85 x 50 mm, with at cut-section a whitish-grey homogenous appearance (**Figure 2A**). Dissection of the peri-pancreatic fat revealed 7 lymph nodes from 3 to 13 mm diameter. Extensive sampling of the tumor was performed for further histopathological analysis. Histopathology showed proliferation of a homogenous population of small ovoid basophilic/amphophilic cells organized in nests, cords and trabeculae inside a thick fibrous capsule (**Figure 2B**), within a irregular hyalinized stroma (**Figure 2C**), negative at Congo Red staining (**Figure 2D**). The mitotic rate remained under 1 mitosis per 10 high-power fields (hpf) and there was no evidence of atypia or tumor necrosis and capsular invasion. Immunostaining revealed strong CD56 and synaptophysin positivity (**Figures 2E, F**, respectively), heterogenous chromogranin positivity (**Figure 2G**) and weak diffuse positivity of neuron-specific enolase (NSE) (**Figure 2H**). The proliferative index Ki-67 remained lower than 2% (**Figure 2I**). Weak and focal cytoplasmic staining of insulin was noted in tumor cells (**Figure 2J**). According to the 2019 WHO classification, the final established diagnosis was “functional well-differentiated pancreatic neuroendocrine tumor of low grade (G1)/insulinoma” (17). Histopathological examination of lymph nodes was otherwise normal. After a 16-month follow-up period after tumor removal, the patient weighted 54 kg (weight loss: 4.2 kg). Insulin, proinsulin, and C-peptide levels as well as CBGM returned within normal ranges. The glycated hemoglobin (HbA1c) and chromogranin A have not been dosed in blood during hospitalization. The patient didn’t address any specific complaint, and no disease recurrence and/or metastasis were observed. A ^68^Ga-DOTATATE-PET scan was performed 6 months after surgery, which came back normal.

## 3 Discussion

Insulinomas are very rare neuroendocrine tumors leading to insulin hypersecretion and hypoglycemia (1, 18). The definition of “giant insulinomas” has been related in the literature in some cases as “insulin-secreting tumors measuring approximately 9 cm of major axis” (19–21). Occurrence rates remain exceptional, with very few cases reported in the literature. In 2013, a comprehensive literature review and report of three cases by Callacondo et al. described the occurrence of 35 cases of giant insulinomas, of which 50% were metastatic to the liver (22). Overall, the sex-ratio was 1.5 (21M/14F), and most tumors exhibited low proliferative indexes and low mitotic rates and features of an amyloid stroma (22). Since the comprehensive review of literature by Callacondo et al. in 2013, apart from the presented case, 9 other cases were reported in the literature (**Table 1**). The sex-ratio of 1.3 (4M/3F) in the cohort was similar to the one observed in the literature review by Callacondo et al. (22). Furthermore, 30% of cases had metastases at time of diagnosis, similarly to the previous review which showed 54% of metastatic recurrence, higher than the usual rates for average-sized insulinomas (1, 22). Previous studies have shown that 18F-fluorodeoxyglucose PET-scan (PET-FDG) could predict the prognosis and be correlated to the histological grade of insulinomas before surgery, thus allowing for better post-operative follow-up (30, 31).The use of 18F-fluorodeoxyglucose PET-scan in a preoperative setting could help to discriminate G1/2 tumors from G3 tumors (30). Furthermore, tumors positive at PET-FDG tend to be larger and more aggressive than negative tumors (30). Data concerning PET-FDG remain scarce or absent in most tumors defined as “giant insulinoma”, however one can hypothesize that giant insulinomas being aggressive tumors (22/44 cases with liver or lymph node metastases), PET-FDG metabolism could be higher in giant insulinomas. In our case, PET-FDG was indeed positive, which suspected malignant insulinoma in our patient. Nevertheless, the 16-month post-operative follow-up showed the absence of metastatic disease, and tumor histology revealed a low-grade (G1) neuroendocrine tumor. Regardless of tumor size, the diagnostic suspicion of malignant insulinoma raises concern for careful monitoring of metastatic disease, especially liver and lymph node metastases, using imaging techniques such as PET-FDG and ^68^Ga-DOTATATE-PET scan to predict prognosis and detect small-sized metastatic sites (10, 32).

**Table 1:** Clinicopathologic features of pancreatic giant insulinomas reported in the literature since 2013.

Author (Ref)	Sex/Age, y	Duration of Symptoms/Main Symptoms and Signs	Pancreatic Location	Size, cm	Pathological findings/Immunohistochemical Study	Metastases/Recurrence	Surgery	Metastases Treatment	Survival
Ielpo et al. (23)	F/57	4 mo/confusion, prosopagnosia	Tail	14 x 10 x 6	“poorly differentiated endocrine carcinoma”, chromogranin +, insulin +, Ki-67>20%	None	DP, S	None	6 y, NDR
Ielpo et al. (23)	F/63	1 y/lumbar pain, anxiety	Head	8 x 6 x 6.5	“well-differentiated neuroendocrine tumor, chromogranin -, insulin -, glucagon -, Ki-67 <20%”, amyloid stroma (60%)	None	PD	None	11 mo, NDR
Fenech et al. (24)	F/76	U/hypoglycemia, neuroglycopenia (Whipple’s triad)	Tail	16 (diameter)	“neuroendocrine tumor, low mitotic count (2/10 hpf), local infiltration of the adipose tissue”	None	DP, S	None	U
Karavias et al. (25)	F/75	6-mo/lightheadedness, diaphoresis	Tail	17 x 15 x 7	“pancreatic insulinoma with malignant potential, insulin +, CEA +, high mitotic count (10 mitoses/10hpf), vascular invasion, tumor necrosis, lymph node and omentum invasion	Liver	DP, S, lAG, lN	None	5 y, NDR
Di Martino et al. (26)	F/49	U/confusion, low consciousness	Head/Tail	21 x17 x 13	“neuroendocrine tumor, Ki-67 10% (G2), high mitotic count (15 mitoses/10 hpf), invasion of peri-pancreatic tissues, 2/15 metastatic lymph nodes)”	Liver, Axillary, Mediastinal	TP, S, PG, C	Radiofrequency ablation	3 y, NDR
Vasikasin et al. (27)	M/15	2-mo/lightheadedness	Tail	12 (diameter)	“neuroendocrine tumor, Ki-67 1-2%, low mitotic count (3-4 mitoses/10hpf), no invasion”	None	DP, S	None	6-mo, NDR
Okada et al. (28)	M/44	U/hypoglycemic symptoms MEN1 (Adrenocortical adenoma, Parathyroid adenomas, Pheochromocytoma, Pituitary adenoma)	Tail	18 (diameter)	“PanNET, Ki-67 1.8%, G1, chromogranin +, synaptophysin +, CD56 +, insulin+” Metastases: “PanNET, Ki-67 4.2%, G2, chromogranin +, synaptophysin +, CD56 +, insulin+”	Liver, Adrenal Glands	DP, S, C, rAG, CTR	Surgical resection	4 y 4-mo, NDR
Ueda et al. (19)	M/71	2-y/impaired consciousness	Head (Uncinate process)	13.5 x 12 x 8	“neuroendocrine carcinoma, chromogranin +, insulin +, glucagon +, somatostatin +, Ki-67 2-5%.”	Adipose tissue, Duodenum, Lymph Nodes	TP	Surgical resection	2-y 3-mo, NDR
Lakha et al. (29)	M/70	U/hunger, anxiety, blurred vision	Head/Tail	15.4 (diameter)	Unresectable tumor (bulky)	None	None	None	U
Tarris et al. (Current case)	F/38	3-mo/malaise, fatigue, fainting	Tail	8.8 x 7.3	“well-differentiated neuroendocrine tumor, G1, low mitotic count (1 mitosis/10 hpf) chromogranin +, synaptophysin +, insulin +, Ki-67<2%”	None	None	None	16-mo, NDR

M, Male; F, Female; y, year; mo, month; DP, distal pancreatectomy; S, Splenectomy; C, Cholecystectomy; TP, Total Pancreatectomy; lAG, left Adrenal Gland resection; lN, left Nephrectomy; PG, Partial Gastrectomy; DP, Duodenopancreatectomy; CTR, Cutaneous Tumor Resection.

In 2016, in the case reported by Okada et al., genetic analysis revealed deletions at codons 83-84 in exon 2 of the *MEN1* gene, which could be incidental or could be related to the etiopathogenesis of giant insulinoma (28). Scientific data emphasizes on the many variations of the *MEN1* gene possibly leading to MEN1 syndrome, which could be family-specific (more than 1,300 MEN1 gene mutations) and showing variable phenotypic spectrum even in identical monozygotic twins (33). Genetic factors, but also epigenetic and environmental factors might contribute to the favoring appearance of giant insulinomas. Mouse models of insulinoma such as cell-line based xenografts (CDXs) or even genetically engineered mouse models (GEMs) might bring new insights into the etiopathogenesis of giant insulinomas (34).

To pursue further, data obtained from mouse models could be confirmed by constituting an international cohort of patients suffering from giant insulinomas, with multi-centric data collection and sampling for further genomic and proteomic analysis. Recent whole-exome sequencing studies in series of average-sized insulinomas demonstrated the role of a T372R substitution in the *YY1* gene as being characteristic of late-onset insulinomas (35). More recently, a whole-genome sequencing study performed in 84 functional insulinomas and 127 non-functional neuroendocrine tumors (NF-PanNETs) showed differential mutational profiles in both tumor subtypes: insulinomas had Copy Number Variation (CNV) amplifications and lacked CNV deletions while CNV-neutral insulinomas exhibited an elevated rate of *YY1* mutations (36). In contrast, NF-PanNETs with CNV alterations and additional *DAXX/ATRX* mutations could exhibit a higher risk of relapse within the first 2-years (36).

Nevertheless, the pathophysiological mechanisms underlying the occurrence of giant insulinomas remain unknown, due to very few sporadic cases worldwide. One hypothesis might be formulated for the explanation of the occurrence of giant insulinomas: exacerbated stimulation of the pathways and/or increased transcriptional activity of the genes involved in the pathogenesis of average-sized insulinomas, under environmental pressure, might explain the important size of these tumors. For example, the activation of the mammalian target of rapamycin (mTOR) pathway might explain overgrowth and potential transformation in giant insulinomas (37). Giant insulinomas could be considered as poor insulin-producing tumors exhibiting slow growth over a period of years without causing symptoms until large size is reached (22). The tail of the pancreas might be an ideal localization for giant insulinomas, as there is more space for tumor growth without any mass effect in a long period of time (22). Alternatively, another hypothesis explaining the occurrence of giant insulinomas might be a unique alternate molecular pathway for tumor growth, cell proliferation, and development of an “amyloid-like” stroma, especially since the amyloid stroma originates from the secretion of amyloid islet pancreatic polypeptide (38). The possible change in phenotypic features between NFPanNETs and insulinomas in the course of tumor growth might explain the growth of large NFPanNETs thus becoming functional (22). Moreover, the focal expression of insulin might be in favor of this hypothesis (22). Cases of grade 1/2 NFPanNETs transforming from a “non-functional” to a “hormone-secreting” phenotype have been previously described in the literature (39, 40). We can hypothesize that hormone secretion and transformation during disease progression could result from the differentiation of intra-tumoral pluripotent stem cells into an “insulin-secreting” phenotype, especially under the influence of pituitary hormones, as previously described in pituitary adenomas (41).

In conclusion, giant insulinomas are very rare tumors showing unique histopathological features, with very scarce knowledge about their pathogenesis. “Giant” insulinomas have a higher recurrence of metastasis, malignancy, and tissue invasion than smaller tumors, therefore indicating the need for careful follow-up to avoid the appearance of metastatic recurrence, especially metastatic liver disease. Further international integrated studies involving transcriptomic analysis and cell culture of giant insulinomas might explain the pathogenesis of these overgrowing tumors, usually presenting with a worse prognosis and requiring risky surgical procedures for safe and complete removal.

## 4 Materials and methods

The resection specimen was handled for gross examination according to standard guidelines (42). The tumor was weighted, measured in its three dimensions, then formalin-fixed before sampling with surgical margins and adjacent pancreatic parenchyma. Formalin-Fixed Paraffin-Embedded (FFPE) tissue blocks were processed in a Tissue-Tek Autotec a120 automaton (Sakura Finetek, Japan). Four-µm thick sections were performed using a Leica microtome. Slides were then stained by HES using a Tissue-Tek Prisma automaton (Sakura Finetek). Antibody detection was performed on 4-µm thick slides in a Dako Omnis automaton (Agilent Technologies). Antibodies used for the diagnosis were directed against CD56 (clone 123C3, ready to use – Agilent Technologies - USA), Chromogranin A (clone LK2H10, 1/200 – Esbe Scientific), Ki-67 (clone Mib-1, ready to use – Agilent Technologies - USA), Synaptophysin (clone DAK-SYNAP, ready to use – Agilent Technologies - USA), Insulin (clone BSB-42, 1/250 – BioSB - USA) and NSE (clone BBS/NC/VI-H14, ready to use – Agilent Technologies - USA). Counterstaining was performed using Hematoxylin (Agilent Technologies - USA). Slide mounting was performed on a Tissue-Tek Film automaton (Sakura Finetek - Japan). Slide digitization was performed on a Nanozoomer 2.0 HT slide scanner (Hamamatsu Photonics - Japan).

## Data availability statement

The raw data supporting the conclusions of this article will be made available by the authors, without undue reservation.

## Ethics statement

Ethical review and approval was not required for the study on human participants in accordance with the local legislation and institutional requirements. The patients/participants provided their written informed consent to participate in this study. Written informed consent was obtained from the participant/patient(s) for the publication of this case report.

## Author contributions

Writing and editing: GT, LM, J-MP, BB, AR. Resources: A-CL, GT, LM, BB, J-MP, AR. HA, PR, KG, RL. Investigation: GT, LM, J-MP, BB, AR, HA, PR, RL, KG. All authors contributed to the article and approved the submitted version.
